# The effects of drift and selection on latitudinal genetic variation in Scandinavian common toads (*Bufo bufo*) following postglacial recolonisation

**DOI:** 10.1038/s41437-020-00400-x

**Published:** 2021-02-09

**Authors:** Filip Thörn, Patrik Rödin-Mörch, Maria Cortazar-Chinarro, Alex Richter-Boix, Anssi Laurila, Jacob Höglund

**Affiliations:** 1 Animal Ecology, Department of Ecology and Genetics, Uppsala University, Uppsala, Sweden; 2 Present Address: Department for Bioinformatics and Genetics, Swedish Natural History Museum, Stockholm, Sweden; 3 Present Address: Department of Zoology, Stockholm University, Stockholm, Sweden

**Keywords:** Evolutionary genetics, Genomics, Genetic variation

## Abstract

Clinal variation is paramount for understanding the factors shaping genetic diversity in space and time. During the last glacial maximum, northern Europe was covered by glacial ice that rendered the region uninhabitable for most taxa. Different evolutionary processes during and after the recolonisation of this area from different glacial refugia have affected the genetic landscape of the present day European flora and fauna. In this study, we focus on the common toad (*Bufo bufo*) in Sweden and present evidence suggesting that these processes have resulted in two separate lineages of common toad, which colonised Sweden from two directions. Using ddRAD sequencing data for demographic modelling, structure analyses, and analysis of molecular variance (AMOVA), we provide evidence of a contact zone located between Uppland and Västerbotten in central Sweden. Genetic diversity was significantly higher in southern Sweden compared to the north, in accordance with a pattern of decreased genetic diversity with increasing distance from glacial refugia. Candidate genes under putative selection are identified through outlier detection and gene–environment association methods. We provide evidence of divergent selection related to stress response and developmental processes in these candidate genes. The colonisation of Sweden by two separate lineages may have implications for how future conservation efforts should be directed by identifying management units and putative local adaptations.

## Introduction

One of the most challenging tasks in modern population genetics is to distinguish between migration, selection, and drift when studying divergence among recently separated populations and species (Hahn 2019). Patterns of contemporary genetic variation along environmental gradients are shaped by the interplay between neutral processes, such as genetic drift, and environmentally mediated and spatially heterogenous natural selection. When these environmental gradients occur over large geographical scales such as latitudinal and altitudinal clines, neutral and adaptive processes can be difficult to tease apart as they result in similar patterns of divergence (Vasemägi 2006; Savolainen et al. 2013; Hoban et al. 2016). Latitudinal gradients are also characterized by complex demographic histories such as founder events and bottlenecks stemming from range contractions and expansions linked to periods of glaciation, which have influenced patterns of genetic variation that we observe today (Hewitt 2000, 2004). In general, genetic diversity is expected to be highest at the centre of a species distribution and decrease towards the margins (Eckert et al. 2008; Guo 2012). However, as genetic diversity also decreases towards the poles, it is further expected to be lower along latitudinal gradients independent of expectations from the central-marginal theory alone (Guo 2012).

During the last glacial maximum (LGM), 23–18 Kya, northern Europe was covered by glacial ice that rendered the region undesirable, and most European animal and plant species had retreated to glacial refugia at lower latitudes in Europe and western Asia (e.g. Petit et al. 2003; Hewitt 2004; Schmitt 2007; Recuero et al. 2012; García-Vázquez et al. 2017; Kühne et al. 2017; Wielstra et al. 2017). Hence, the genetic landscape of species inhabiting northern Europe has been shaped by both selection and drift in the different glacial refugia and along the different recolonisation routes (Eckert et al. 2008; Hampe and Petit 2005; Guo 2012; Weir et al. 2016). Allopatric populations may have experienced divergent selection within their respective ranges. Furthermore, populations at the edge of range expansions likely suffered loss of within-population genetic variation due to bottlenecks, resulting from founder events due to the increased influence of genetic drift in small populations (Eckert et al. 2008). This has led to a general pattern of genetic diversity being negatively correlated with distance from the glacial refugia (Hewitt 2000). However, admixture events occurred when populations once in allopatry met during range expansions, resulting in regions with higher genetic diversity (Petit et al. 2003).

The Scandinavian Peninsula provides an interesting setting for studying the phylogeography of postglacial colonisation. Colonisation routes to the peninsula were available from the south through a land bridge between present day Denmark and Sweden and from the north through Finland (Herman et al. 2014). This has led to some species having genetically distinct populations along the latitudinal range of Scandinavia as reflected in their recent evolutionary history (Hewitt 2004). Examples of such distinct genetic clusters as a result of different colonisation routes are found among both animals and plants (Taberlet et al. 1995; Knutsson and Knutsson 2012; Günther et al. 2018; Rödin‐Mörch et al. 2019).

Here, we investigated the glacially influenced evolutionary history of common toads, *Bufo bufo* along a latitudinal gradient spanning from southern to northern Sweden. There is conflicting evidence regarding common toad phylogeography of Scandinavia. Garcia-Porta et al. (2012) placed Scandinavian toads in a single cluster together with central European populations. However, no samples from northern Scandinavia were included in that study. In contrast, Dufresnes and Perrin (2015) suggested a colonisation of the Scandinavian Peninsula from a refugium located in the Balkans via the east across Finland. In addition, large-scale studies targeting the common toad species complex have not captured the northern distribution of the species range, which has left Scandinavia unresolved (Recuero et al. 2012; Arntzen et al. 2013). However, a study of mitochondrial lineages along the Norwegian coast suggested dual colonisation routes, from the north and the south, respectively, (Tuncay et al. 2018).

Amphibians are presently the most threatened vertebrate taxon and phylogeographic surveys are becoming more important to evaluate their conservation (Wake and Vredenburg 2008; Nielsen et al. 2019). If two separate lineages have colonised Scandinavia from two different directions, this has important applications for practical conservation. In common toads, class IIB major histocompatibility complex (MHC) diversity has been found to decrease from south to the north in Sweden (Meurling 2019). Furthermore, infection experiments with the chytrid fungus *Batrachochytrium dendrobatidis* have shown higher mortality in northern individuals compared to southern (Meurling 2019). Hence, the lower MHC diversity in the northern populations imposes a threat of local extinction and loss of evolutionary potential if there is a genetic difference between northern and southern Scandinavia common toad populations. If two separate lineages have colonised Scandinavia from two different directions, there may be reasons for dividing the Swedish populations into separate conservation units in order to preserve evolutionary potential (Moritz 1994; Forest et al. 2007).

The aim of this study was to unravel the evolutionary history since the last glaciation of the common toad as well as to investigate the patterns of genetic diversity along the latitudinal gradient across Sweden. We sampled 12 populations divided equally between four regions along the 1400 km latitudinal gradient. We used a double digest restriction-site-associated DNA (ddRAD) sequencing protocol to obtain genome-wide single-nucleotide polymorphism (SNP) data from the different populations. Using an outlier-based method as well as gene–environment associations, we identified candidate SNPs under putative selection. Using neutral SNPs, we used population genomic methods to unravel the postglacial evolutionary history of these populations. We also investigated patterns of genetic diversity found along the latitudinal gradient. Through demographic model selection, we obtained the best fitting model describing the recolonisation of the Scandinavian peninsula. If the recolonisation of Scandinavia was as a result of a single southern postglacial colonisation route, in accordance with Scandinavian common toads belonging to the central European complex, we expect to find a pattern of decreasing genetic diversity from the south to the north along the latitudinal gradient (Garcia-Porta et al. 2012; Recuero et al. 2012; Arntzen et al. 2013) as previously supported by findings of MHC haplotype diversity (Meurling 2019). An alternative hypothesis is that common toads may have colonised Scandinavia from two different directions as shown in a number of other species, and also suggested by previous work on mitochondrial diversity in populations along the Norwegian coast (Hewitt 2000; Tuncay et al. 2018; Rödin‐Mörch et al. 2019). Genetic diversity may still be lower in the north compared to the south in the event of a two directional recolonisation scenario, as genetic diversity will decrease with distance to refugia (Hewitt 2000). Therefore, we implement multiple approaches in order to determine the most likely recolonisation scenario.

## Methods

### Sample collection and DNA extraction

**Fig. 1:**
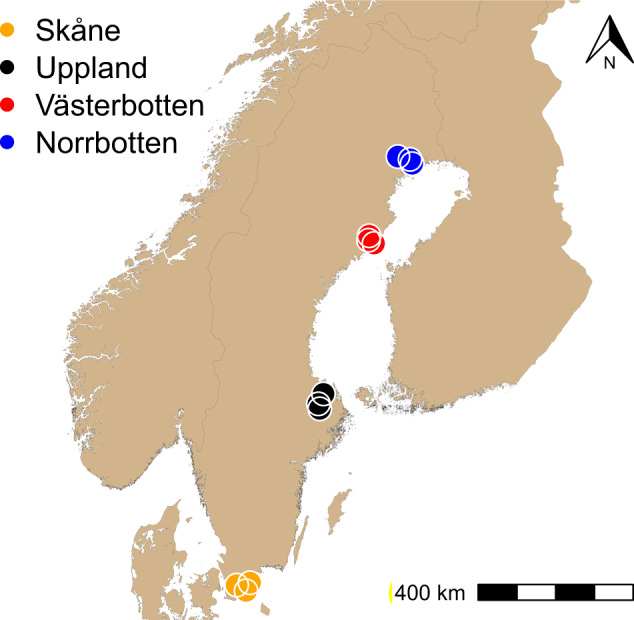
The location of the sample populations of common toads. The study includes 12 populations from four different regions: Norrbotten (blue), Västerbotten (red), Uppland (black), and Skåne (orange).

Adult common toads (*n* = 240) were collected from 12 different populations in Sweden along a 1400 km long latitudinal gradient in April–May 2015 (Fig. 1, Table S1). Three populations were sampled for 20 individuals per population in each of the four regions Skåne, Uppland, Västerbotten, and Norrbotten. A tissue sample was taken from the hind leg webbing of each toad in the field. DNA extraction was carried out using the DNeasy blood and tissue Qiagen kit by following the manufacturer’s protocol. The extracted DNA was stored at −20 °C until January 2019 and was assessed for potential fragmentation during storage using gel electrophoresis on 1.5 % agarose gel. Ten samples from each population were selected for barcoding and sequencing (*n* = 120), based on the visual inspection of the electrophoresis gel making sure that sample DNA was not fragmented.

### Library preparations and sequencing

ddRAD library preparation was conducted following the protocol of Johansson et al. (2017). Each sample was digested using the restriction enzymes MseI and SbfI-HF for 16 h at 37 °C. Adaptors for dual indexing barcoding were ligated at the cut sites; every sample received a unique combination of SbfI-HF and MseI adaptors. The samples were then purified with magnetic bead cleaning (AMPure XP) before and after amplification through polymerase chain reaction (PCR). Amplification was carried out by two sequential PCR reactions. The first PCR was run at four replicates for each sample (thermal profile: 98 °C for 30 s; 20 cycles of: 98 °C for 20 s, 60 °C for 30 s, 72 °C for 40 s; final extension at 72 °C for 2 min). All replicates from the same sample were then pooled and ran for a second PCR (thermal profile: 98 °C for 3 min, 60 °C for 2 min, 72 °C for 2 min). DNA fragments from the same individual received a unique dual index barcode from the combination of the Illumina primer barcode and the SbfI-HF adaptor, this made identification of each fragment possible after sequencing.

All the samples from the final PCR product were pooled at equal volumes and ran on agarose gel. A gel slab containing DNA fragments with a length between 300 and 500 bp was cut out of the gel for size selection. The DNA was extracted from the gel using a gel extraction kit (QIAquick MinElute Gel Extraction Kit). The DNA product from the gel extraction was purified through magnetic beads cleaning (AMPure XP). The library was sequenced at SciLife Laboratory in Uppsala on an Illumina NovaSeq 6000 SP flow cell with a paired end read length of 150 bp.

### SNP calling

The raw reads were processed with the program STACKS 2.41 (Catchen et al. 2013). The reads were demultiplexed to individuals and quality checked in STACKS. Reads where the raw phred score was below ten were discarded.

The demultiplexed reads were de novo assembled using STACKS before SNPs were called. Only the first SNP from each read was called. The pipeline parameters were optimised to obtain the highest number of SNPs without too much loss of coverage. The optimisation process was carried out by modifying the core parameters in the pipeline (Paris et al. 2017). The parameters that were modified were the maximum number of gaps allowed between nucleotides within samples (-M), the number of mismatches allowed in the alignment between samples (-n) when constructing the catalogue of all consensus loci, and the number of populations each SNP needed to be present in to be called (-p) (Tab S6). Parameters not mentioned were kept as default. The final dataset used a combination of these parameters that produced the most SNPs without loss in coverage. The final dataset was obtained with -M 2, -n 3, -p 10, as this combination produced the highest number of SNPs without loss of coverage (Tab S6). All the SNP calling was carried out using Uppsala Multidisciplinary Centre for Advanced Computational Science computer cluster.

### Filtering SNPs under putative selection

SNPs under putative selection were obtained using the principal component analysis (PCA)-based method pcadapt, implemented in R (Luu et al. 2017; R Core Team 2019). An SNP dataset is a multivariate space with as many dimensions as there are SNPs. As SNPs are correlated to each other, it is possible to decompose the multivariate space into *k* principal components (PCs), which explain a proportion of variation in the dataset from multiple SNPs in a single dimension. Population structure corresponds to differences in shared genetic variation between individuals; individuals who are more related have more shared genetic variation amongst them relative to individuals who are less related. As each PC is a single dimension, SNPs correlated to each other are closer on the PC and can therefore be used to infer population structure. Pcadapt utilizes this by calculating vectors of *z*-scores for each SNP derived from regressions between *j* SNPs and *k* PCs (for equations see Luu et al. 2017). Outliers are then obtained by calculating the Mahalanobis’ distance of all the *z*-score vectors and converting them into *p* values. Based on a scree plot of the 20 first PCs, we decided to calculate the Mahalanobis’ distance for *k* = 4 as these PCs captured most of the population structure (Fig S3). The R package qvalue was used to extract outliers with a *p* value below 0.05 (Storey Lab 2019). Outlier SNPs are under putative selection as they are associated with population structure and assumed to be candidates for local adaptation.

Gene–environment associations were also used to find putatively selected SNPs with latent factor mixed models (LFMM) (Caye et al. 2019). In the R package LFMM, we used growing season length for each population as a fixed effect and used four latent factors corresponding to the population structure displayed in the PCAs to identify SNPs under putative selection. Growing season length was defined as the number of days annually when the average temperature reaches 5 °C or higher (Table S8). We used temperature data averaged over the period 31 December, 2005–31 December, 2015 obtained from (http://opendata-download-metobs.smhi.se/explore/#, accessed 20 November 2016). The LFMM function was run using the ridge penalty and an FDR of 0.05.

Both pcadapt and LFMM rely on multiple statistical tests to identify SNPs under selection. The use of multiple statistical tests increases the risks of false positives, i.e. significant results produced by random chance (Hoban et al. 2016). We corrected for the false positive discovery by applying a false positive discovery rate of 0.05 in both methods.

The outliers were removed from the SNP dataset to retain only neutral markers, thus analyses that assume that markers are evolving under neutrality were less prone to errors. The read fragments containing the selected SNPs were queried against the Megablast, blastn, and blastx databases (minimum similarity 60%, Megablast and blastn *e*-values below 1 × 10^−10^, and blastx *e*-value below 1 × 10^−5^). Summary statistics were calculated for the neutral dataset to investigate connectivity between populations, calculations of summary statistics were included in the STACKs pipeline (pairwise *F*_ST_, nucleotide diversity, expected/observed heterozygosity, expected/observed homozygosity, and inbreeding coefficient). Allelic richness was calculated using the R package PopGenReport (Gruber and Adamack 2019).

### Isolation by distance

Isolation by distance was tested with a Mantel test. The Mantel test was performed using a pairwise-*F*_ST_ matrix that was standardised using the *F*_ST_/(1 − *F*_ST_) and a distance matrix of the natural logarithm of the geographic distances in kilometres between each population (Rousset 1997). The test was run for 99,999 permutations using Pearson correlations in the R- package vegan (Oksanen et al. 2013).

### Cluster analysis

Cluster analysis was carried out to infer sample ancestry using the R package TESS3r, which uses least-square approximations (Caye et al. 2018). TESS3r incorporates geographical distance between samples in the cluster algorithm, which when left unaccounted for may bias the estimation of admixture proportion. Separate Q matrixes were created for 1–12 different ancestral populations (*K*) using the neutral SNP dataset; the cluster algorithm was repeated 50 times for each value of *K* and averaged across runs. The best number of ancestral populations was chosen based on the cross-validation score plots (Fig. 4a).

### Demographic modelling

The software fastsimcoal 2.6 was implemented to find the demographic scenario that best fitted our data. Fastsimcoal 2.6 is a likelihood method that builds on the sequential Markovian coalescent model (Excoffier and Foll 2011; Excoffier et al. 2013). Fastsimcoal 2.6 obtains an estimated likelihood by simulating a multi-dimensional folded site frequency spectrum (mSFS) under a provided demographic scenario. By comparing the simulated estimate mSFS with the observed mSFS model fit is evaluated. An mSFS consists of the distribution of allele frequencies derived from multiple populations. A folded mSFS is used when the ancestral states are not known.

We generated the observed mSFS from 14,285 SNPs that were obtained from STACKS by running the populations module using the parameter set -M 3, -n 2, -p 12 while also removing the SNPs found to be under putative selection in pcadapt and LFMM. We decided to increase the number of populations (*p*) each SNP needed to be represented in to reduce the presence of missing data when running Fastsimcoal 2.6. We constructed models of demographic histories for the establishment of each region from an ancestral population. By using regions instead of populations for the demographic histories, we reduce the number of parameters needed to be estimated, which makes interpretation of the models easier while also reducing computation time. Using regions instead of populations is valid as the PCA plots and TESS3r cluster analysis all show the populations from each region being clustered together. The python module easySFS was used to generate the observed mSFS (Overcast 2019). In the module, we further restricted our data by downsampling the number of individuals in each region from 30 to 7. This decreases frequency of rare sites (as suggested from https://github.com/isaacovercast/easySFS).

We tested a total of six different models (Fig. 5). The M1 model describes a single-direction postglacial colonisation route from north to south. The M2 model describes a single-direction postglacial colonisation route from south to north. Models M3–M6 describe bidirectional colonisation routes after the LGM; the models differ in whether the Uppland region has been established from the northern regions or from the southern regions. Migration over a contact zone was also tested in models M4 and M6.

Fastsimcoal 2.6 draws parameter values from distributions during the simulation of estimated mSFS. A uniform distribution was used to obtain the parameter values for effective population sizes (N_e_) and a logarithmic uniform distribution was used for the population resizing parameters. The migration and time since divergence were given as complex parameters, leading to migration being a function of population size, and time to divergence was estimated relative to previous time splits in the model. A generation time of 3 years was used for the simulations and a mutation rate of 7.7e−10 as estimated for the western clawed frog *Xenopus tropicalis* and High Himalaya frog *Nanorana parkeri* (Sun et al. 2015). No estimated mutation rate exists for the common toad.

Each model was run for 100,000 simulations for 50 replications, after which the best run from each model was selected based on the difference between observed and estimated maximum likelihood. Akaike’s information criterion (AIC) calculations were used for model selection between the best replicates of each model; complex parameters were not scored multiple times when calculating AIC.

### Analysis of molecular variance (AMOVA)

AMOVA was carried out to test for differences between the northern and southern regions. The test was implemented in the R package poppr (Excoffier et al. 1992; Kamvar et al. 2014). The AMOVA tests if the genetic differences within populations, between populations, and between the northern and southern regions are different from random expectations. We decided to group the northern region (Västerbotten and Norrbotten) and the southern regions (Skåne and Uppland) into a northern and a southern lineage, based on the results from the demographic models, PCA, and cluster analysis. Significance testing of the AMOVA results was carried out using a randomization test with 999 permutations from the R package ade4 (Dray and Dufour 2007; Bougeard and Dray 2018).

## Results

### Differentiation outliers and gene–environment associations

After the de novo assembly of ddRAD sequences, a total of 17,636 SNPs were obtained from the SNP calling pipeline. The mean coverage over loci was 53.4×.

**Fig. 2:**
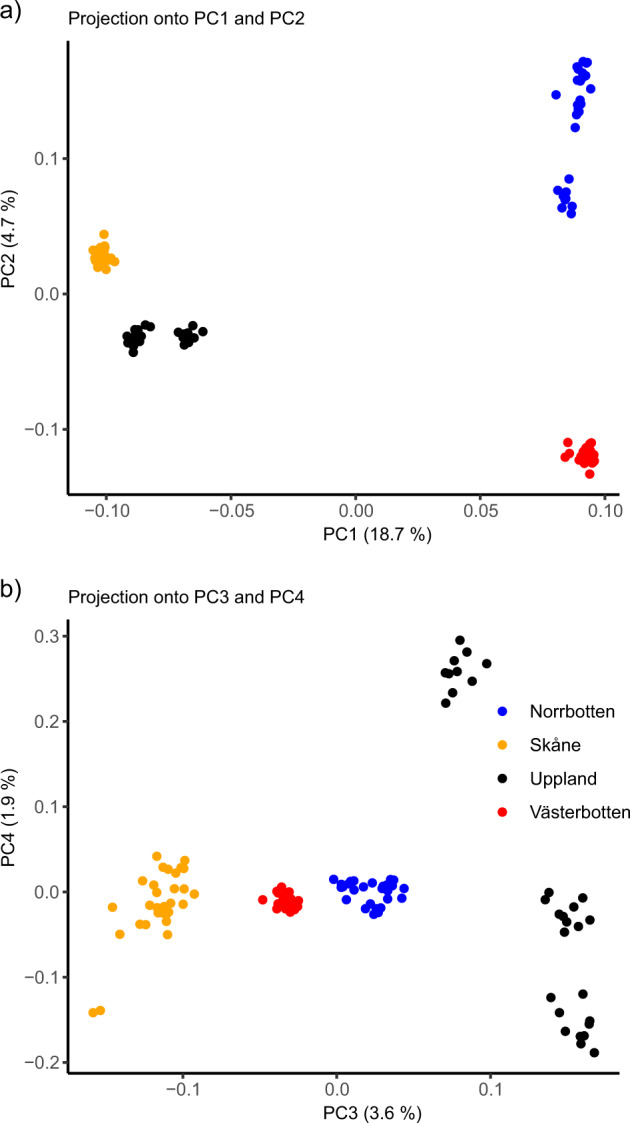
Population structure of Scandinavian common toads. PCA of individuals of common toads projected on to PC1 and 2 (**a**) and PC3 and 4 (**b**) using the unfiltered SNP dataset. Samples obtained from: Norrbotten (blue), Västerbotten (red), Uppland (black), and Skåne (orange).

The outlier analysis on the first four *PC* axes found 472 SNPs under putative selection and the gene–environment associations found 233 SNPs to be under putative selection. A total of 84 loci were identified by both methods and thus we detected a total of 621 SNPs under putative selection and were removed to obtain a dataset of neutral variation containing 17,015 SNPs. Isolation by distance was confirmed by a Mantel test (*r* = 0.754, *p* < 0.0001). A visualisation of the association is presented in Fig S4. The PCA plots from the outlier analysis separated the two northernmost regions from the southern regions along PC1 (Fig. 2a). The difference between the two most northern regions was revealed along PC2 (Fig. 2b). PC3 displayed the difference between the southern regions (Fig S1a) and PC4 separated the populations within Uppland (Fig S1b).

### Genetic diversity and population structure

**Fig. 3:**
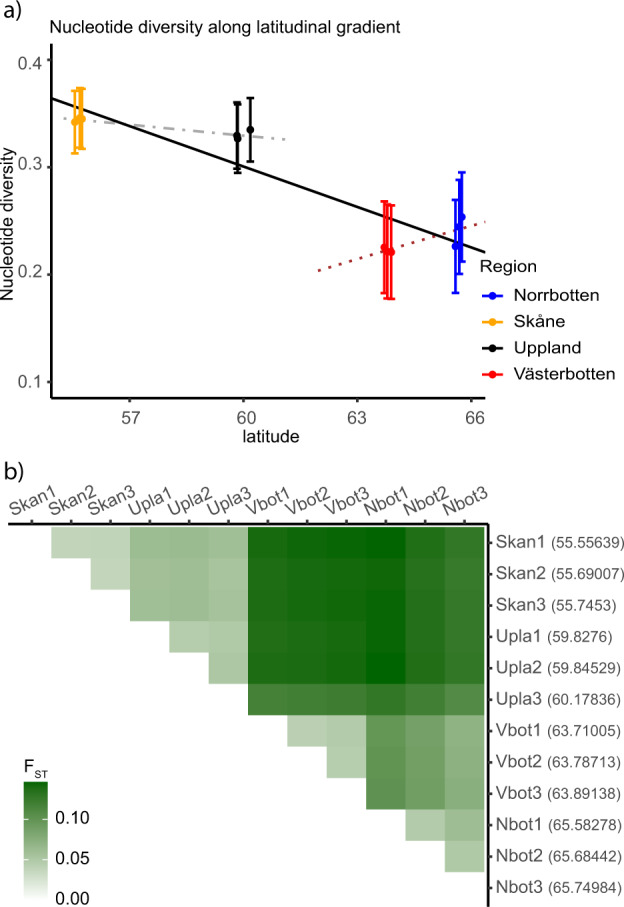
Patterns of genetic diversity in Scandinavian common toads. **a** Nucleotide diversity for common toad populations plotted along the latitudinal gradient. Regression for Skåne and Uppland in light grey (*p*=0.0145, *r*^2^=0.81, slope=0.00103), regression for Västerbotten and Norrbotten in dark grey (*p*=0.0674, *r*^2^=0.608, slope=−0.00319), and regression for all populations in black (*p*<0.0001, *r*^2^=0.806, slope=1.054). **b** Heatmap of pairwise *F*_ST_ between common toad populations, dark green corresponds to higher values of *F*_ST_. The populations are referred to: Nbot1–3 from Norrbotten, Vbot1–3 from Västerbotten, Upla1–3 from Uppland, and Skan1–3 from Skåne. The latitude of each population is given inside the brackets on the *y*-axis. The numbering of the populations refers to their placement along the latitudinal gradient, populations with 3 as suffix are located the furthest north in their region.

Neutral genetic diversity was lower at higher latitudes as nucleotide diversity (π) and expected heterozygosity (H_e_) were lower for the populations in Norrbotten and Västerbotten (Welch two-sample *t*-test. π: *p* < 0.001 (df = 8.21), H_e_: *p* < 0.001 (df = 8.28), Fig. 3a). Note that the Västerbotten populations tended to have the lowest diversity. All populations had inbreeding coefficients (*F*_is_) close to 0, there was no significant difference between expected and observed homozygosity (*χ*^2^ test: *χ*^2^ = 132, df = 121, *p* value = 0.2329). Summary statistics for observed and expected homozygosity and heterozygosity, nucleotide diversity, inbreeding coefficients, and allelic richness are available in Table S7. Pairwise *F*_ST_ was highest between the northern and the southern populations, the highest difference being found between Skan1 and Nbot1 (*F*_ST_ = 0.149, Fig. 3b). AMOVA revealed significant difference at all levels. The variance within populations, between populations, and between lineages all differed from random expectations (Table 1). Most of the variation was found at population level. Visualisation of the AMOVA results, significance table, and covariance table are available in Supplementary Material (Fig S2, Table S2).

**Table 1:** AMOVA significance testing table.

Test	Obs	Std.Obs	Alter	*p* value
Variation within populations	1799.786	−36.862	Less	0.001
Variation between populations	301.157	38.098	Greater	0.001
Variation between lineages	793.956	7.332	Greater	0.002

Significance is based on a randomization test with 999 permutations. Hierarchy for the AMOVA was populations within lineages for common toads. Lineage was defined as the northern regions (Västerbotten + Norrbotten) and the southern regions (Skåne + Uppland). “Obs” is observed variance at hierarchical levels, “Std.Obs” is the observation in the randomized data, and “Alter” refers to if the observed value is less or greater than random expectations for the alternative hypothesis.

**Fig. 4:**
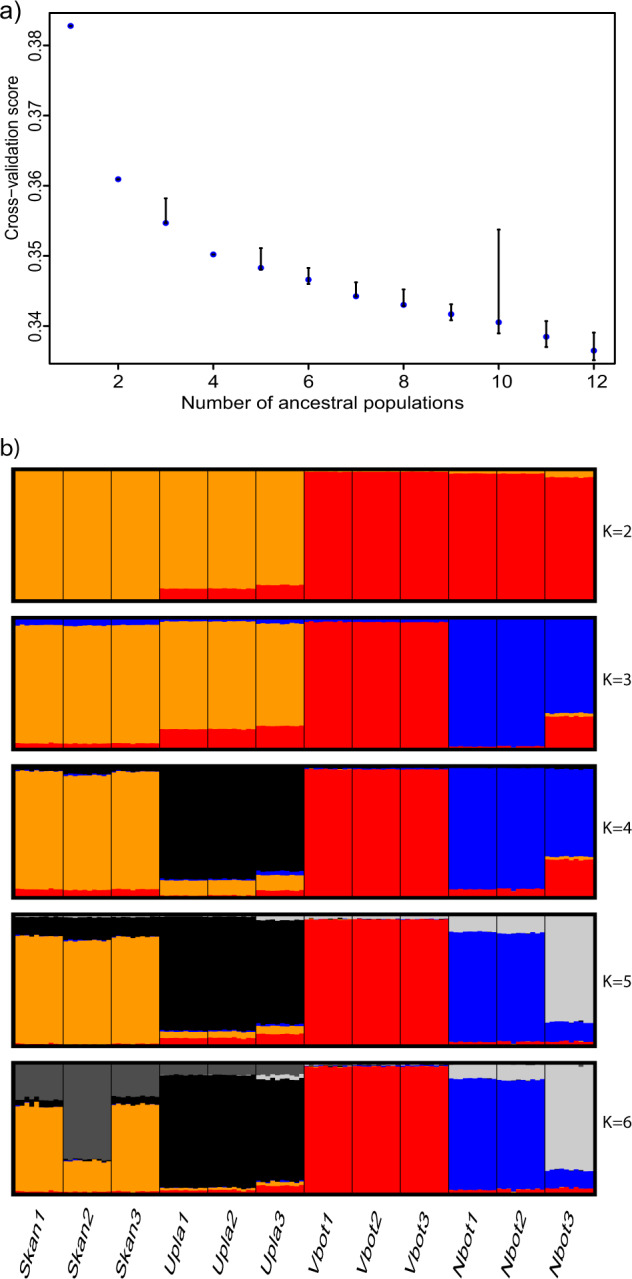
Ancestral populations from TESS3R. **a** Cross-validation score from TESS3r plotted against number of ancestral populations (*K*) of common toads in Scandinavia. Cross-validation is performed through calculating root mean-squared errors for a subset of loci. The best *K* is found where the cross-validation score starts to plateau. **b** Cluster analysis bar plot for values of *K* between 2 and 6, implemented through TESS3r. Populations of common toads are grouped by region and are ordered by increasing latitude, each bar is one individual.

Based on the cross-validation, the score plot started to level out at 2–4 ancestral populations (*K*); the TESS3r analysis revealed the best number of ancestral populations to be in the range of 2–5 distinct genetic clusters (Fig. 4). For *K* = 2, Skåne and Uppland formed one cluster and Västerbotten and Norrbotten another. Populations from Skåne and Uppland clustered together and Västerbotten and Norrbotten were separated when the number of ancestral populations was assumed to be 3. At *K* = 4, populations clustered by region. At *K* = 5 the Norrbotten population furthest north (but west of the Luleå river) separated from the other populations in that region (Fig. 4b).

### Demographic model histories

**Fig. 5:**
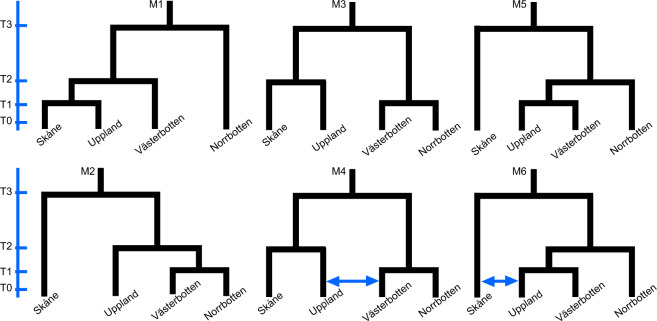
Demographic scenarios used to simulate mSFSs in fastsimcoal 2.6 for common toad in Scandinavia. A single postglacial route of Scandinavia is modelled in M1 and M2 and a dual postglacial route is modelled in M3–M6. Arrows indicate migration events and population divides occur at T3–T1. Populations are resized after each population divide.

Six different demographic histories were tested in fastsimcoal 2.6 (Fig. 5). The best fitting model was M4, which modelled a southern and a northern lineage meeting somewhere between Uppland and Västerbotten with secondary contact through migration (Fig. 5; M4). M4 fitted the data best based on the delta maximum likelihood value as well as having the lowest AIC (Table 2).

**Table 2:** Model selection results from demographic models describing postglacial colonisation of Scandinavia by the common toad.

Models	EstimateMaxLikelihood	DeltaLikelihood	AIC	DeltaAIC	AICWeights
M1	−30725.353	3625.085	61470.706	6392.396	0
M2	−30201.732	3101.464	60423.464	5345.154	0
M3	−27549.444	449.176	55118.888	40.578	1.54E−09
**M4**	**−27527.155**	**426.887**	**55078.31**	**0**	**0.999999998**
M5	−28497.032	1396.764	57014.064	1935.754	0
M6	−27930.695	830.427	55885.39	807.08	5.56E−176

Columns from left to right: model identity, estimated maximum likelihood of the model, the delta maximum likelihood between the simulated and observed mSFS, AIC, delta AIC (difference between the lowest AIC of the best fitting model and the model), and AIC weights (AIC weights are to be interpreted as conditional probabilities for each model). The best fitting model is given in bold.

### SNPs under selection

The 621 RAD tags that contained SNPs found to be under putative divergent selection were queried against nucleotide and protein databases using BLAST in an effort to annotate these sequences. For the pcadapt outliers: 36 separate sequences had hits in blastx, 34 sequences had hits in blastn, and 55 sequences had hits in Megablast. For the LFMM outliers: 20 sequences had hits in blastx, 27 sequences had hits in blastn, and 12 sequences had hits in Megablast. Several of these candidate genes under putative selection were of particular interest as they relate to development and immune responses. Among these were zinc finger protein 341 (Znf341), vascular endothelial growth factor receptor kdr-like (kdrl), DnaJ heat shock protein family (Hsp40) member C28 (dnajc28), death-associated protein 3 (DAP3), and nuclear factor of activated T cells 5 (NFAT5). *e*-values, hit length, and similarity for all hits within the cut-off intervals are found in Tables S3–S5.

## Discussion

The main goal of this study was to investigate the patterns of genetic diversity along the latitudinal gradient of Sweden on the Scandinavian Peninsula, as well as to unravel the evolutionary history of the common toad in Scandinavia since the last glaciation. Previous studies have made no distinction between common toads in Scandinavia, which would suggest a single direction for postglacial colonisation (Garcia-Porta et al. 2012; Recuero et al. 2012; Arntzen et al. 2013). However, the present study did not support this single direction of postglacial colonisation but instead provided evidence of bidirectional postglacial routes. This was supported by the division of the southern regions (Skåne and Uppland) and northern regions (Västerbotten and Norrbotten) on PC1 in the PCA, the grouping of the southern populations and northern populations in the cluster analysis for *K* = 2, and best fitting demographic model, M4, being a bidirectional colonisation scenario. The study found that genetic diversity was lower in the northern regions, as nucleotide diversity and expected heterozygosity were lower relative to southern regions. This lower diversity in the north is in accordance with a negative relationship between genetic diversity and distance to refugia as the southern lineage probably used a more western refugium, while the refugium for the northern lineage was located further to the east (Hewitt 2000). Bidirectional postglacial routes were also supported by a recent mitochondrial study of toads along the coast of Norway (Tuncay et al. 2018).

### Genetic diversity and demographic history

Nucleotide diversity was lower for Norrbotten and Västerbotten (northern populations) compared to Skåne and Uppland (southern populations), which is to be expected from a longer postglacial colonisation route utilised by the populations now inhabiting the northern regions. In the northern regions, Västerbotten shows slightly lower nucleotide diversity compared to Norrbotten (dark grey regression line in Fig. 3a), providing additional support for the northern regions having entered Scandinavia from a postglacial colonisation route via Finland. The relatively high nucleotide diversity in Uppland can be explained by this region being close to the contact zone between the northern and southern genetic clusters, as genetic diversity is predicted to increase at secondary contact (Petit et al. 2003). Support for this contact zone is also present in the best fitting demographic scenario, M4 (Fig. 5). The pattern of genetic diversity we observe across Sweden may also be explained by the central-marginal latitudinal hypothesis (Eckert et al. 2008; Guo 2012): the higher genetic diversity in the southern populations can be explained by them being closer to the centre of the common toad’s European distribution and the northern populations having lost diversity by being on the margin of the distribution, as well as loss of genetic diversity from the latitudinal expansion. This explanation would fit both single and bidirectional colonisation routes. However, with the evidence of bidirectional colonisation, diversity in Swedish common toads is more likely explained by the differences in distance from the putative refugia of the two colonisation routes (Hewitt 2000). Nevertheless, central-margin patterns and distance from refugia are not mutually exclusive and together can produce complex patterns (Guo 2012; Cortázar-Chinarro et al. 2017; Rödin‐Mörch et al. 2019). No signs of extensive inbreeding were found in any of the populations as inbreeding coefficients were low (Table S7).

The Uppland populations did not create a distinct single regional cluster in the PCA (Fig. 2a, b; Fig S1a, b), instead one Uppland population (Upla3) was separated from the other two populations (Upla1 and Upla2). This separation may be due to the different environmental characteristics of the sampling locations within Uppland. The population that clustered separately from the other two was located in a clear nutrient-poor forest lake, whereas the other two Uppland populations were located in more eutrophic waters. This could give rise to different genetic profiles in the populations by local adaptation. Alternatively, this separation may also be explained by gene flow from the northern regions southward. As the Upla3 population is the furthest north in Uppland, it may experience a larger contribution from the northern lineage. Differences in pairwise *F*_ST_ between the Uppland populations and the northern populations support the theory of increased gene flow. The population Upla3 had slightly lower difference in pairwise *F*_ST_ with the northern populations as compared to Upla1 and Upla2 (Fig. 3b). There is also a possibility that movement across the Bothnian Bay from Finland to Sweden has occurred during the range expansion of the common toad after the LGM. This type of migration route has been shown in the European common adder *Vipera berus*, where a contact zone was found between Västerbotten and central Finland (Carlsson et al. 2004). The presence of migration across the Bothnian Bay could in future studies be investigated by including samples from Finland.

The demographic analysis indicate that the putative hybrid zone lies between Uppland and Västerbotten, as has been shown in studies of *Rana arvalis* (Knopp and Merilä 2009; Rödin‐Mörch et al. 2019). The PCA plot of PC1 and PC2 (Fig. 2a) as well as the TESS3r bar plot for *K* = 2 (Fig. 4) also supported this divide between Uppland and Västerbotten. This placement of the contact zone may be common across widespread taxa in Scandinavia and the contact zone for several vertebrate species has been placed there (e.g. Taberlet et al. 1995; Bensch et al. 1999; Andersson et al. 2004; Rödin‐Mörch et al. 2019).

### Candidate genes under putative selection

The BLAST results for the reads that contained differentiation outliers were mapped to their related *PC* axes. Outliers that are related to PC1 divergence are SNPs that are associated with the clustering between the northern and the southern regions. Outliers mapped to PC2 and PC3 are associated with the population clustering within the northern region and within the southern region, respectively. Lastly, outliers mapping to PC4 are associated with the clustering of populations within Uppland. In the BLAST results, we found the Znf341 and vascular endothelial growth factor receptor kdrl genes to be associated with PC1, thus being under putative selection between the northern and southern regions. Znf341 is a central regulator of immune homoeostasis in humans and may have a similar function in the amphibians (Frey-Jakobs et al. 2018). The vascular endothelial growth factor receptor (kdrl) is important during the development of the olfactory system in amphibians during metamorphosis (Pozzi et al. 2006). The putative selection on vascular endothelial growth factor receptors may be an adaptation to the shorter growth season in the north. However, it was not identified to be under selection by the growing season association method. The dnajc28 and DAP3 genes were found to be associated with PC3, which indicate putative selection between Uppland and Skåne. The heat shock protein (Hsp40) is related to protein translation and contributes to thermal adaptation in *Drosophila melanogaster* (Qiu et al. 2006; Carmel et al. 2011). DAP3 is important in the control of apoptosis (Wazir et al. 2015). Both Hsp40 and DAP3 could be of importance during metamorphosis, which would indicate adaptive differences in development between the southern regions. During amphibian metamorphosis apoptosis occurs during the remodelling of tissues (Ishizuya-Oka et al. 2010). Hsp40 was also found to be under selection in the growing season length association method. The NFAT5 is under putative selection between the Uppland populations as it maps to PC4. NFAT5 regulates osmotic stress response in humans and may have a similar function in the toads, potentially by increasing fitness in coastal populations (Neuhofer 2010). However, more targeted studies are needed to truly identify genes under selection, which is why throughout the paper these candidate genes are referred to as under putative selection.

### Conservation implications

As two major genetic clusters of common toads are present in Sweden, presumably as a result of different postglacial colonisation routes, an argument can be made for these clusters to be seen as separate conservation units. Per definition, separate conservation units need to be significantly diverged at nuclear loci and reciprocally monophyletic for mtDNA alleles (Moritz 1994). With the evidence of divergence between the southern and northern lineages of common toad presented in this study, which is confirmed by the significant difference between lineages in the AMOVA test, a strong argument for significantly diverged nuclear loci is present for the common toad in Sweden. Potential conservation efforts should treat the lineages as separate conservation units.

The results in this study suggest that after the LGM, two separate lineages of common toad colonised Sweden from two directions. This type of bidirectional colonisation of the Scandinavian Peninsula have been found in other animal taxa such as the *R. arvalis*, *V. berus*, and *Ursus arctos* (e.g. Taberlet et al. 1995; Carlsson et al. 2004; Knopp and Merilä 2009; Rödin‐Mörch et al. 2019). The best fitting demographic scenario models a bidirectional colonisation with southern regions having a separate colonisation route from northern regions. The best model also identifies migration between the regions Uppland and Västerbotten, which indicate a contact zone being present somewhere between these regions. A future study targeting the gradient between Uppland and Västerbotten with denser sampling would be needed to identify the location of the contact zone. We found that genetic diversity is higher in the southern regions compared to the northern ones, which is in accordance with a pattern of decreased genetic diversity with increasing distance to refugia as well as from the expectations from the central-marginal latitudinal hypothesis. Finally, colonisation of Scandinavia by two separate lineages of common toad has produced genetic differences along the latitudinal gradient. Future conservation efforts will have to account for potential discrepancies between the northern and southern common toad populations. Future studies should aim to address these potential discrepancies.

## Supplementary information

Supplemental Material

Table_S3

Table_S4

Table_S5

## Data Availability

The datasets generated and/or analysed during the current study are available at Dryad (https://datadryad.org/stash/share/23JUFlzI6kHdQA-tSq-wOe23VKX0wttzFI_dCwhVYQ8).
