# Supplementary material for: The effects of drift and selection on latitudinal genetic variation in Scandinavian common toads (Bufo bufo) following postglacial recolonisation
Source: Heredity (Edinb). 2021 Feb 9;126(4):656–67. doi: 10.1038/s41437-020-00400-x (PMC8115047; doi:10.1038/s41437-020-00400-x)
Supplement: Supplementary file 1 — Supplemental Material [file 41437_2020_400_MOESM1_ESM.pdf]

Supplemental Material

Tables S1, S2, S6, S7 and S8 are present in this document. Tables texts for tables S3, S4 and S5 are present in this document. Tables S3, S4 and S5 are available in:

Table_S3.xlsx

Table_S4.xlsx

Table_S5.xlsx

Figures S1, S2, S3 and S4 are present in this document.

Tables S1-S8

Table S1. Names, regions and coordinates of sample populations of common toad.

**Population**	**Region**	**Latitude**	**Longitude**
Skan1	Skåne	55.55639	13.95864
Skan2	Skåne	55.69007	13.48483
Skan3	Skåne	55.7453	14.15416
Upla1	Uppland	59.8276	17.66872
Upla2	Uppland	59.84529	17.59817
Upla3	Uppland	60.17836	17.85447
Vbot1	Västerbotten	63.71005	20.39697
Vbot2	Västerbotten	63.78713	20.12291
Vbot3	Västerbotten	63.89138	20.15539
Nbot1	Norrbotten	65.58278	22.31932
Nbot2	Norrbotten	65.68442	22.21299
Nbot3	Norrbotten	65.74984	21.60165

Table S2. AMOVA Components of covariance. Hierarchy for the AMOVA was Populations within Lineages for common toads in Scandinavia. Lineages were defined as the northern regions (Skåne + Uppland) and the southern regions (Västerbotten + Norrbotten) grouped separately.

	**Sigma**	**%**
Variations Between Lineage	793.9555	27.42602
Variations Between populations Within Lineage	301.1572	10.40303
Variations Within populations	1799.7860	62.17095
Total variations	2894.8987	100.00000

Table S3. Blastx candidate gene hits. Samples marked in grey were found in both by the gene association LFMM method and by Pcadapt.

Table S4. MegaBlast candidate gene hits. Samples marked in grey were found in both by the gene association LFMM method and by Pcadapt.

Table S5. Blastn candidate gene hits. Samples marked in grey were found in both by the gene association LFMM method and by Pcadapt.

Table S6. Parameter sets for the optimisation runs with STACKs and resulting coverage and number of SNPs.

Run	M	n	p	coverage gstack	#SNPs
a	2	2	12	52.6x	14151
b	2	3	12	53.4x	14818
c	3	2	12	52.7x	14189
d	3	3	12	52.9x	14307
e	3	4	12	53.4x	14491
f	4	2	12	52.9x	13648
g	4	3	12	53.0x	13760
h	2	3	10	53.4x	17636
i	3	4	10	53.4x	17431
j	4	3	10	53.0x	16579

Table S7. Summary statistics calculated from neutral SNPs. From left to right: Pop ID = population identification; Obs Het = Observed heterozygosity; Obs Hom = Observed Homozygosity; Exp Het = Expected Heterozygosity, Exp Hom = Expected Homozygosity; Pi = Nucleotide diversity; F_{is} = Inbreeding coefficient, Allel rich = Sum of allelic richness.

Pop ID	Obs Het	Obs Hom	Exp Het	Exp Hom	Pi	F_{is}	Allel rich
Skan1	0.33676	0.66324	0.32449	0.67551	0.34201	0.02287	26667.93
Skan2	0.33515	0.66485	0.32816	0.67184	0.34592	0.03595	26860.60
Skan3	0.34201	0.65799	0.32732	0.67268	0.34506	0.01775	26808.48
Upla1	0.32284	0.67716	0.31243	0.68757	0.32947	0.02664	26403.32
Upla2	0.32526	0.67474	0.30966	0.69034	0.32663	0.01332	26260.53
Upla3	0.32216	0.67784	0.31765	0.68235	0.33490	0.04147	26588.80
Vbot1	0.22113	0.77887	0.21390	0.78610	0.22544	0.02043	22800.15
Vbot2	0.22309	0.77691	0.21028	0.78972	0.22161	0.00792	22484.73
Vbot3	0.22079	0.77921	0.20957	0.79043	0.22090	0.01153	22441.99
Nbot1	0.22507	0.77493	0.21470	0.78530	0.22633	0.01401	22739.14
Nbot2	0.24719	0.75281	0.23181	0.76819	0.24444	0.00474	23222.80
Nbot3	0.25212	0.74788	0.24054	0.75946	0.25368	0.01369	23767.18

Table S8. Growth season length measurements and weather stations used to obtain climate data. Growing season length was defined as the number of days annually when the average temperature reaches 5°C or higher. Temperature data averaged over the period 31 December 2005–31 December 2015.

Pop ID	Growing season length	Weather station
Skan1	254.6	Lund
Skan2	254.6	Lund
Skan3	254.6	Lund
Upla1	214.9	Uppsala Aut
Upla2	214.9	Uppsala Aut
Upla3	214.9	Uppsala Aut
Vbot1	170.2	Umeå Flygplats
Vbot2	170.2	Umeå Flygplats
Vbot3	170.2	Umeå Flygplats
Nbot1	161.3	Luleå Flygplats
Nbot2	161.3	Luleå Flygplats
Nbot3	161.3	Luleå Flygplats

Figures S1-4

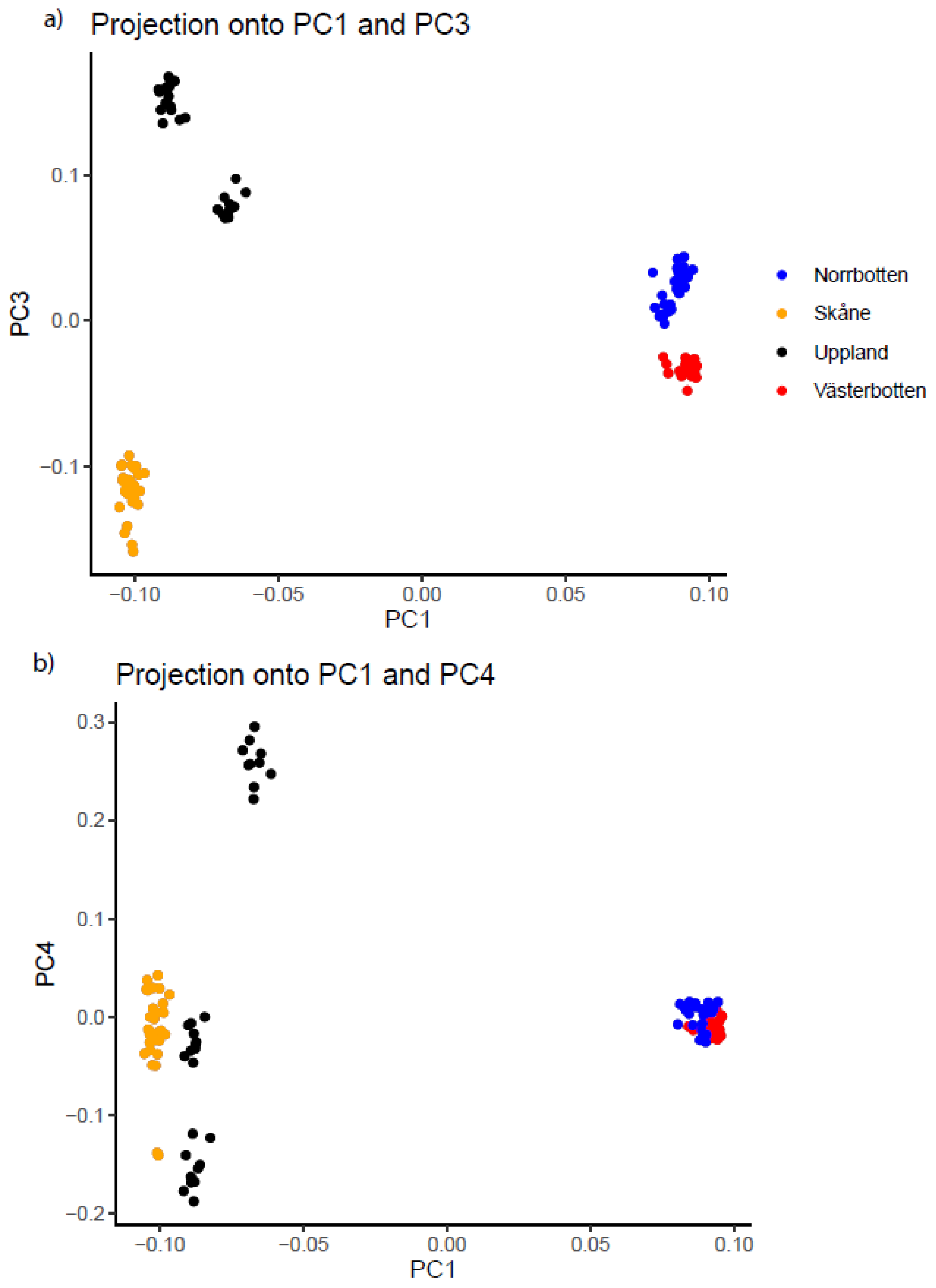

Fig S1. PCA of individuals of common toad projected on to PC 1 and 3 (a), and PC 1 and 4 (b) using the unfiltered SNP data set. Norrbotten (blue), Västerbotten (red), Uppland (black), and Skåne (orange). Clear separation between regions visible in both projections.

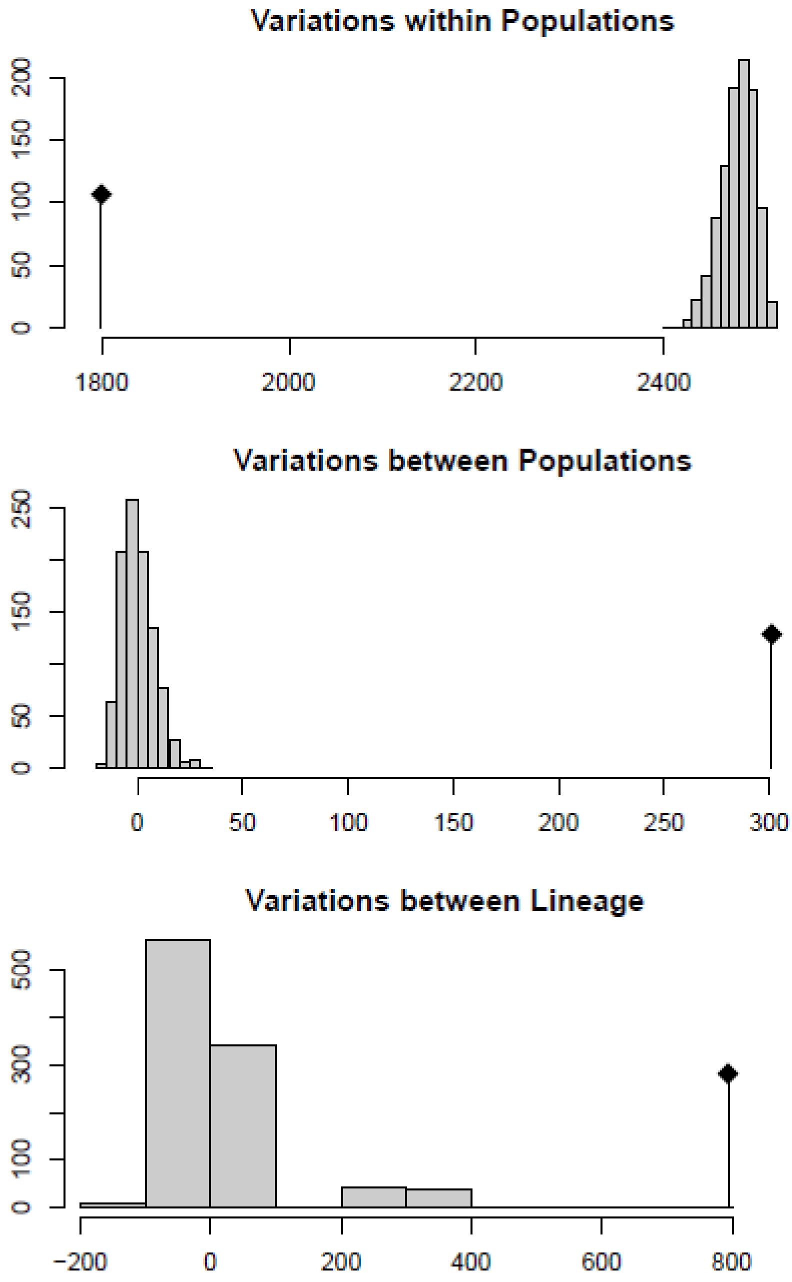

Fig S2. Histograms visualising the randomisation test for the AMOVA results. Hierarchy for the AMOVA was Populations within Lineages. Lineages were defined as the northern regions (Västerbotten + Norrbotten) and the southern regions (Skåne + Uppsala) grouped separately.

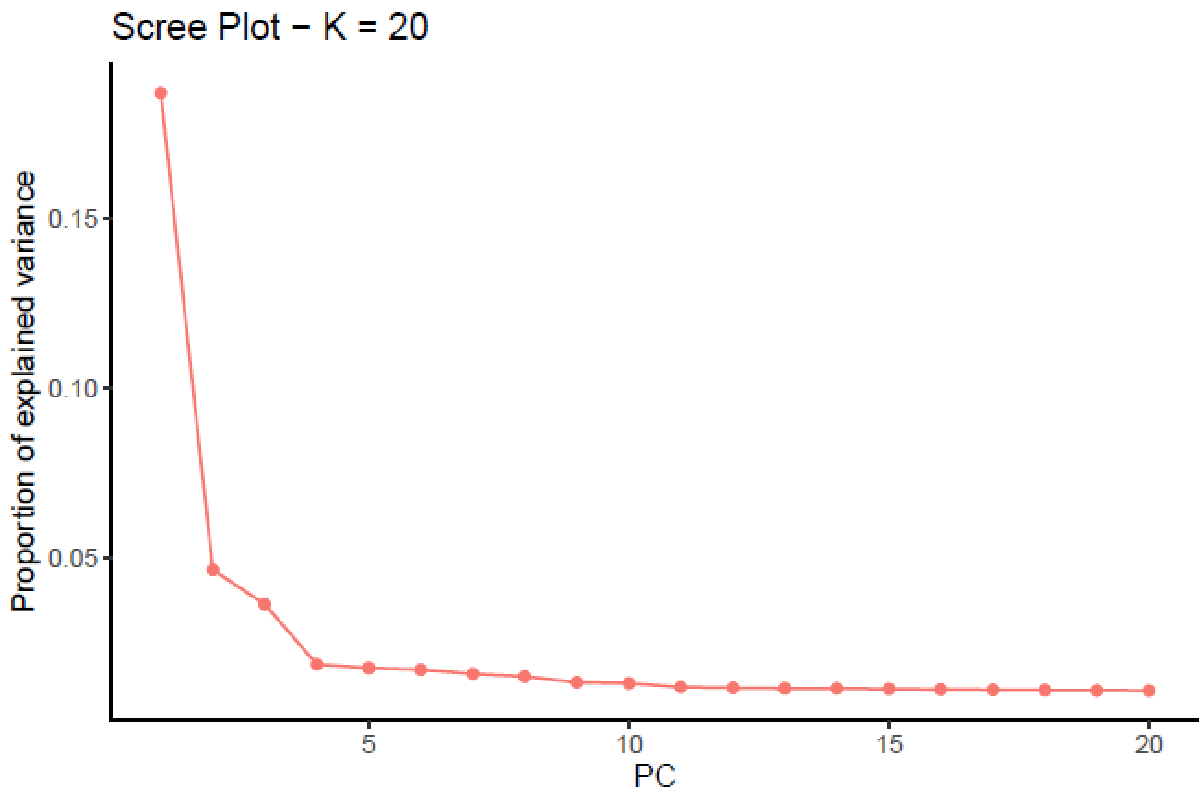

Fig S3. Scree-plot displaying the optimal value for K for the Pcadapt outlier detection method. Ancestral populations on the x-axis and proportion of explained variance on the y-axis. The optimal value is obtained where the curve plateaus. i.e K=4.

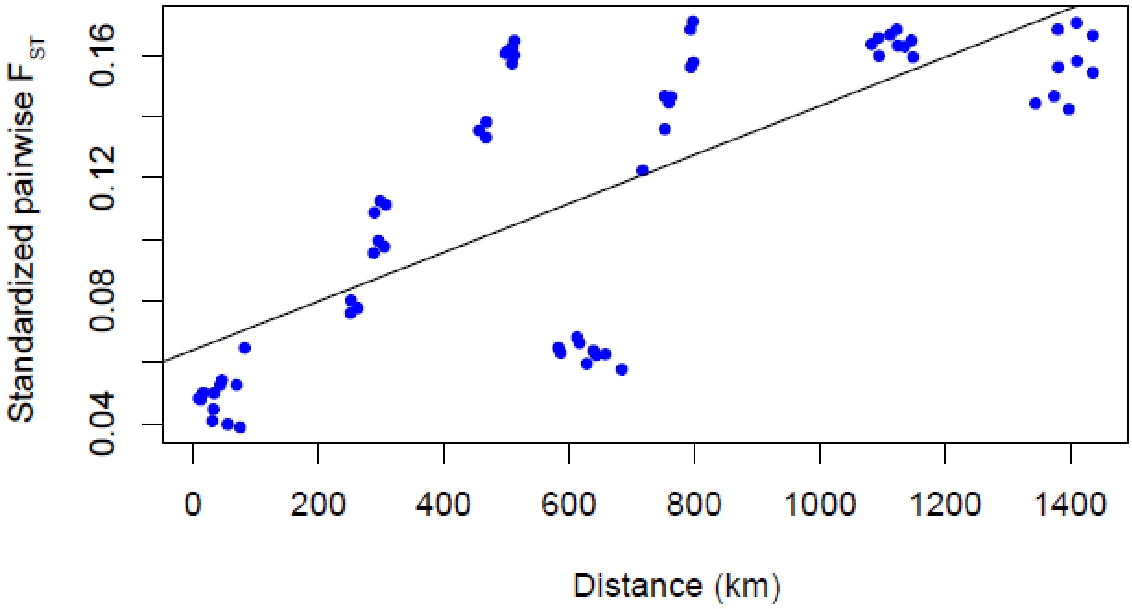

Fig S4. Association between geographic distance and pairwise F_{ST}. Distance in km on x-axis and standardised F_{ST} on the y-axis.
